# Pre‐pragmatic language use in toddlerhood: Developmental antecedents, aetiological factors, and associations to autism

**DOI:** 10.1002/jcv2.12135

**Published:** 2023-01-11

**Authors:** Maja Rudling, Ana Maria Portugal, Sven Bölte, Terje Falck‐Ytter

**Affiliations:** ^1^ Department of Psychology Development and Neurodiversity Lab Uppsala University Uppsala Sweden; ^2^ Department of Women's and Children's Health Center of Neurodevelopmental Disorders (KIND) Centre for Psychiatry Research Karolinska Institutet Stockholm Sweden; ^3^ Curtin Autism Research Group School of Allied Health Curtin University Perth Western Australia Australia; ^4^ Swedish Collegium for Advanced Study Uppsala Sweden

**Keywords:** autism spectrum disorder, decontextualized language, language development, pragmatic language, social communication, twin analysis

## Abstract

**Background:**

Pragmatic language is key for adaptive communication, but often compromised in neurodevelopmental conditions such as autism spectrum disorder (ASD). Decontextualized language—to talk about events and things beyond here and now—develops early in childhood and can be seen as a pre‐pragmatic ability. Little is known about the factors that contribute to decontextualized language use in toddlers and whether these are different from factors contributing to general language development.

**Methods:**

We studied longitudinal associations between parent‐rated core language and non‐verbal socio‐communicative abilities at 14 months of age, and decontextualized language use at 24 months of age in children with typical and elevated likelihood of ASD (total *N* = 303). Using twin modelling, we also investigated genetic and environmental contributions on decontextualized language and grammar use in two‐year‐old twin pairs (total *N =* 374).

**Results:**

Core language ability was a strong predictor of later decontextualized language use in both children with and without an elevated likelihood of ASD. In contrast, social communication was only a significant predictor of decontextualized language use for children with low levels of core language. This pattern was specific to decontextualized language, and not replicated in prediction of concurrent grammatical ability. Further, there was a large genetic influence on decontextualized language at 2 years of age, which mostly overlapped with the genetic influences on grammatical ability. Shared environment influences were significant for grammatical ability, but not found on decontextualized language. In children with an elevated likelihood of ASD, decontextualized language use was negatively associated with autistic symptoms.

**Conclusions:**

This study suggests that decontextualized language is developmentally associated with, yet dissociable from, more general language development measured as grammatical ability. Already at 2 years of age, parental ratings of decontextualized language is associated to clinician‐rated symptoms of ASD.

**Key points**

- Pragmatic language is important for everyday life and difficulties with pragmatic language are associated with ASD.
- Despite its theoretical and practical relevance, little is known about the early development of pragmatic language.
- We showed that core language at 14 months predicted decontextualized language use at 24 months, a potential early measure of pragmatics, and that it moderated the association between social communication and decontextualized language.
- Already at 2 years of age, parent‐rated decontextualized language use was associated with clinician‐rated autistic symptoms and with likelihood of ASD, beyond core language ability and non‐verbal general developmental level.
- These results advance our understanding of early pragmatic language use in both typical and autistic development, with potential implication for directed intervention.

## INTRODUCTION

To use language in a socially adaptive way requires a range of abilities. Core language, here defined as vocabulary, phonology, and grammar, is required to put thoughts into words and to linguistically convey meaning. Socio‐communicative abilities are utilized to interpret and adapt to ever‐changing social situations. Conceptually in between core language and social communication is pragmatic ability. This, we use to adapt language to communicate effectively with others, taking others' perspectives into account to form coherent and meaningful utterances given the conversational context. The influential Relevance Theory (Carston, 2002; A. C. Wilson & Bishop, 2019; D. Wilson & Sperber, 2002) proposes that while we use both core language and pragmatic abilities when inferring meaning from often ambiguous linguistic information, these two aspects are still clearly dissociable. In this theory, pragmatics is seen as part of domain‐general processes that are not only separable from the decoding module of core language, but rather functions to infer meaning from communicative information more generally. However, all tests of pragmatic ability put demands on core language ability, and sometimes vice‐versa, making the two aspects hard to empirically differentiate. Potentially as a consequence, core language and pragmatics scores generally correlate strongly and are closely associated developmentally, and empirical support for a clear dissociation between core and pragmatic language has been inconclusive (Happe, 1993; Matthews et al., 2018; A. C. Wilson & Bishop, 2019, 2021a). According to an account put forward by Wilson and Bishop (2019, 2021a) core and pragmatic language abilities may be most closely interrelated in early language development, becoming subsequently more separate as linguistic and social capabilities increase. To advance our understanding of the development of pragmatic ability, an important question is therefore how core and pragmatic language are longitudinally and aetiologically associated in early development. This question has been difficult to study, partly due to the difficulty in measuring pragmatics in a valid way, especially in early language development.

In autism spectrum disorder (ASD), a neurodevelopmental condition characterized by alterations of social communication and behaviours (DSM‐5; American Psychiatric Association, 2013), pragmatic language difficulties are practically universal (Baird & Norbury, 2016; M. Miller et al., 2015; A. C. Wilson & Bishop, 2021b). In contrast, core language abilities vary greatly between individuals with the condition (Kjelgaard & Tager‐flusberg, 2001). Despite the high prevalence of pragmatic language difficulties in ASD, the development of pragmatic and core language in association to symptoms of ASD is not well understood. Such knowledge could be crucial for developing early directed interventions to better support autistic children and their families.

It has been argued that, moving forward, the study of pragmatic development should avoid global measures of pragmatics and instead focus on specific pragmatic abilities (Matthews et al., 2018). One such pragmatic ability is the use of decontextualized language; to talk about perceptually absent referents, such as past or future events, or objects and ideas that are not present (Rowe, 2013; Ucelli, 2009). To do this proficiently, one must linguistically provide the context that is needed for correct references. Cognitively, this requires an understanding of the conversational common ground; socially, it requires sensitivity and adaptation to the listeners understanding and knowledge; and linguistically, it requires a proficient enough syntactic and semantic ability to convey references clearly. Typically, children start using decontextualized language around 1.5–2 years of age (Berglund & Eriksson, 2000b; P. J. Miller & Sperry, 1988; Ucelli, 2009), initially relying on interpretation and co‐construction from conversational partners (Uccelli et al., 2019). Arguably, this makes decontextualized language an early emerging pre‐pragmatic ability, potentially suitable for studies of pragmatic development in young ages. To our knowledge, only one study has focussed on how core language and social communication are associated with the development of decontextualized language use. Miniscalco and colleagues studied 36 children with an ASD diagnosis, and found that imitation at three years of age predicted improvement in decontextualized language use from ages three to four years, whereas core language did not (Miniscalco et al., 2014). However, no comparison group was used, so we cannot conclude if these associations were specific for children with ASD.

In the present study, we had two pre‐registered research questions. First, we studied how early core language and non‐linguistic socio‐communicative abilities at 14 months were associated with decontextualized language use at 24 months of age in children in the general population. Based on the notion of co‐dependency between social and linguistic abilities early in development (A. C. Wilson & Bishop, 2019, 2021a) we expected both core language and social communication in infancy to be important for decontextualized language use in toddlerhood, potentially interacting with each other. Second, we studied how early decontextualized language use was associated to autistic traits in toddlerhood and with likelihood of ASD due to having a first‐degree family member with the condition. Because of the link between pragmatic language and autism later in development, we expected to find an association between decontextualized language use and autism. To investigate these two questions we utilized two longitudinal study samples: Babytwins Study Sweden (BATSS; Falck‐Ytter et al., 2021) and Early Autism and ADHD Sweden (EASE; Falck‐Ytter et al., 2018). The BATSS sample consisted of same‐sex twin pairs. The EASE sample consisted of children with a first‐degree relative with an ASD diagnosis, and a comparison group of children with no first‐degree relatives with the diagnosis, constituting the elevated‐likelihood group and the typical‐likelihood group respectively. In accordance with the analysis plan, developmental antecedents were studied in children from the BATSS and the typical‐likelihood group from EASE, representing the general population. Associations to autism, here measured as autistic traits and likelihood group membership, were studied in children from the EASE sample.

Two secondary analyses were later added to investigate the theoretical dissociation between core and pragmatic language in the general population. Here we did not include the elevated likelihood group from EASE, as we had no specific hypotheses of the association between core and pragmatic language being different for children with elevated likelihood of later ASD diagnosis. First, we tested if the longitudinal association between core language and social communication was specific to decontextualized language use or generalised also to concurrent core language, measured as grammatical language use at 2 years age. Second, utilizing the twin sample, we investigated how aetiological factors were associated with decontextualized language use and early grammatical ability.

## METHODS

### Participants

Participants from the two studies (BATSS and EASE) were included if they had data at both time‐points (14 and 24 months of age). General exclusion criteria for the BATSS and EASE studies were diagnosis of epilepsy or history of fits/convulsions; any known genetic syndrome clearly related to ASD; any known significant, uncorrected vision or hearing impairment; any known significant developmental or medical condition likely to affect brain development; premature birth (prior to week 34 for BATSS, and week 36 for EASE).

#### The BATSS sample

The children in the BATSS sample were recruited from the greater Stockholm area, via the national population registry in Sweden. Eleven children were excluded from the main, analyses and 19 from the twin analysis due to not fulfiling inclusion criteria (seizures at the time of birth, meningitis early in life, and twin‐to‐twin transfusion syndrome). Because measures from twins are not independent, only one, randomly chosen twin from each pair was included in the main analyses (*N* = 178, 49.4% boys). In the twin analysis, both co‐twins were included (*N* = 374, 49.2% boys). The zygosity of twin pairs was estimated based on DNA in saliva (monozygotic twins *N* = 209, dizygotic twins *N* = 165).

#### The EASE sample

The EASE sample (*N* = 125, 52.8% boys) was divided into two groups depending on whether the children had a first‐degree relative with ASD or not. The recurrence rate of ASD in younger siblings of children with the condition is ∼20% (Ozonoff et al., 2011), making the likelihood of ASD elevated in families with an autistic member compared to the general population. Children in *the elevated likelihood group* (*N* = 97, 53.6% boys) were recruited via the EASE project's website, advertisement, and clinical units. They had a sibling or parent with an ASD diagnosis, which was confirmed through clinical interviews and medical records. Children without a first‐degree relative with ASD were considered to have *typical likelihood of ASD*, and these children (EASE typical likelihood group; *N* = 28, 50.0% boys) were recruited from birth records and advertisements.

#### Combined typical likelihood group

Because none of the included variables, except for chronological age (see below), differed significantly between the BATSS (one‐twin) sample and the typical‐likelihood group of the EASE sample (even after correcting for chronological age), we combined these two groups of children to form *the combined typical likelihood group* (*N* = 206, 49.5% boys), representing the general population.

### Measures

#### Swedish early communicative development inventory

The Swedish Early Communicative Development Inventory (SECDI; Berglund & Eriksson, 2000a; Eriksson & Berglund, 1999) is the Swedish version of the Macarthur‐Bates Communicative Development Inventory (CDI; Fenson et al., 1994). It is a questionnaire wherein parents rate early linguistic and social behaviours.

The two main independent variables were extracted from the SECDI Words and Gestures form and collected when the participants were ∼14 months old. The Total Gesture Score was used as a measure of social communication. It consists of 63 1‐point items on five subscales: First Communicative Gestures, Games and Routines, Actions With Objects, Pretending To Be a Parent, and Imitating Other Adult Actions. The Core Language Composite was calculated by combining z‐scores of the scales Comprehension of Sentences and the Comprehension Score to get a broader measure of core language that included both grammar and vocabulary. The z‐scores were extracted from the group means separately for the combined typical likelihood group and the EASE elevated likelihood group when this measure was to be used for the groups separately. In the analyses of associations with group membership (the EASE elevated likelihood and typical likelihood groups) the z‐scores were based on the means of these two groups combined. Comprehension of Sentences consists of 27 common, short phrases that the parent's mark if their child understands (e.g. “clap your hands”). The Comprehension Score measures receptive vocabulary; parents mark which words in a checklist of 370 common words that their child understands. These two measures correlated strongly in our sample, both for the combined typical likelihood group (*r* = 0.76, *p* < 0.001) and the elevated likelihood group (*r* = 0.81, *p* < 0.001).

The main dependent measure, used as a measure of decontextualized language, was the Pragmatics Scale (“How children use language”) of the SECDI Words and Sentences form, collected when participants were ∼24 months old. The last item (whether the child make up names for toys) was excluded, as this item generally decreases the internal consistency of the scale and was excluded from the norming process of the SECDI (Berglund & Eriksson, 2000a). Thus, the scale contained five 0‐to‐2‐scaled items: (1) Does your child ever talk about things that have happened before? (2) Does your child ever talk about things that are going to happen in the future? (3) Does your child ever talk about things that are missing, for example, if a doll has disappeared or about people that are not present? (4) Does your child understand if you ask about something that is not immediately present, that is, if you ask your child to get a toy from another room (5) Does it ever happen that your child picks up or points towards another person's object and speak the name of that person?

The Grammar Scale, of the SECDI Words and Sentences form, consists of six items (maximum 12 points), with questions about the child's use of morphological markers of the possessive, singular, plural, past tense, and supine forms (a common marker of the past tense in Swedish).

#### ADOS‐2

The ADOS‐2 (Lord et al., 2012) is an instrument used to elicit ASD‐associated behaviours in semi‐structured play situations. In the EASE sample, children were evaluated with the ADOS‐2 at ∼24 months of age. Two different modules were used depending on the developmental level of the child: The toddler module and module 2. To compare scores across modules, we used the calibrated severity scores.

#### Background variables

Background information was collected when the children were 5 months. Socio‐economic status (SES) was defined as the highest educational level on a 4‐point scale (averaged between parents in the cases where information on both parents was available; all but 48 participants). The information of whether at least one parent spoke Swedish as their first language was missing from 48 participants in the EASE study, due to study‐protocol changes. Chronological age was defined as the child's age at the time when the SECDI forms were filled in electronically. Within the EASE sample, for 23 participants at the 14‐months time‐point and 11 participants at the 24‐months time‐point, the date of registering the SECDI form was incorrect in the database and was therefore changed to that of the corresponding lab visit.

### Analyses

The analysis plan was pre‐registered in the Open Science Framework (DOI 10.17605/OSF.IO/F7GV8) before any analysis was performed. Hypotheses related to developmental antecedents and links to ASD symptoms and likelihood were pre‐registered, while the specificity‐ and twin analyses were not. Analyses were performed using SPSS version 27 (IBM Corp., Armonk, N.Y., USA), and the PROCESS tool (Hayes, 2013). Significance tests were all two‐tailed with an alpha level of 5%. All variables were checked for violations against parametric tests; kurtosis and skewness of each variable was checked to not extend beyond +/− 1 (see Supporting Information Figure S1‐S7 for plots of the distribution), Cook's distances and individual z‐scores were calculated to identify influential cases and outliers, and distributions of standardized residuals were inspected through Q‐Q plots. Due to non‐normal distribution of predictors, we report bootstrapped confidence intervals and standard errors for correlation and regression analyses (1000 samples; bias corrected and accelerated (BCa) for the main analyses, percentile for the moderation analyses).

Chronological age differed significantly between samples and groups, and the SECDI is not standardized for age. Therefore, in all subsequent analyses we regressed out age from the Core Language Composite and Total Gesture Scores, by using the residuals of regression models where age was used as a covariate and the Core Language Composite and Total Gesture Score respectively was used as dependent variables.

#### Developmental antecedents

In accordance with the pre‐registered analysis plan, this analysis was made using the combined typical likelihood group. Pearson's correlations were calculated between the Pragmatics Scale, Core Language Composite, Total Gesture score and the potential control variables; chronological age at the 24‐months time‐point, SES, sex, and whether Swedish was the first language of either parent (the two latter dichotomous variables were analysed using point‐biserial correlations (pb). The correlations that included the Pragmatics Scale was partial, with chronological age at the 24‐months time‐point as control variable. To investigate the relative contribution of core language and social communication on decontextualized language use we proceeded with a hierarchical regression analysis with the Pragmatics Scale as dependent variable. Variables were entered in three pre‐specified, increasingly complex models: (1) Control variables, (2) The Core Language Composite and Total Gesture Score, (3) The interaction term.

#### Link to symptoms and likelihood of ASD

For the elevated likelihood group, we calculated Pearson's correlations between the Pragmatics Scale and ADOS‐2 scores. The SECDI‐measures were compared between the children with and without elevated likelihood of ASD using one‐way ANOVAs with likelihood group as factor and chronological age (in days) as control variable. To test associations with likelihood of ASD, likelihood group was entered as a main term and a three‐way interaction term with the Core Language Composite and Total Gesture Score in the final model of the main hierarchical regression analysis, with all lower‐level interaction terms.

#### Secondary analyses

##### Specificity analysis

To explore whether the results of the main hierarchical regression analysis was specific to the Pragmatics Scale, we performed the same hierarchical regression analysis as in the main analysis, instead using the Grammar Scale as the dependent variable. This analysis was carried out using the combined typical likelihood sample, to keep it in line with the primary antecedent analysis above.

##### Twin analysis

For the follow‐up twin modelling, we used R software (version 4.0.0) with the OpenMx package version 2.18.1 with NPSOL optimizer (Neale et al., 2016) and full‐information maximum likelihood estimation. Age was regressed from the Pragmatics and Grammar Scales before being entered in the generalized estimating equation (EEG) models by using the residuals of regression models with age as covariate and the Pragmatics and Grammar Scales respectively as dependent variables. Sex was added as a covariate in the models.

Twin models estimate the relative contribution of genetic and environmental factors to the variation in a phenotype and/or to the covariation between phenotypes, by comparing, respectively, the correlation between twins or the cross‐trait cross‐twin (CTCTs) correlations (the correlation between one phenotype for one twin and another phenotype for their co‐twin). By doing this separately for monozygotic twins (MZ; who share 100% of their segregating genetic material) and dizygotic twins (DZ; who on average share 50%), the variation or covariation can be decomposed into additive genetic influences (A; heritability which increases twin similarity), non‐shared environment (E; environmental influences that differ between twins and decrease twin similarity, including measurement error), and shared environment (C; environmental influences that increase twin similarity regardless of zygosity).

First, univariate modelling of the Pragmatics Scale and the Grammar Scale were run by fitting saturated models (testing for the assumptions of equality of mean and variances across twin order and zygosity) and twin models (testing A, C, and E influences). Then, bivariate modelling was run by fitting a saturated model and twin models. For the latter, a correlated factor solution was fitted to examine the genetic/environment correlations between the Pragmatics Scale and the Grammar Scale. The best fitting model was evaluated based on the AIC fit statistic (Akaike information criterion; the lowest value corresponds to the best model). Twin and CTCTs correlations were derived from the constrained saturated bivariate model in which means, variances, and phenotypic and CTCTs correlations were constrained to be equal across twin order and zygosity.

## RESULTS

### Developmental antecedents

Descriptive statistics are reported in Table 1. In accordance with the pre‐registration this analysis was performed on the combined typical likelihood group. The Pragmatic Scale correlated with the Core Language Composite (*r(203)* = 0.41, *p* < 0.001, 95%CI [0.28–0.51]) and Total Gesture Score (*r(203)* = 0.33, *p* < 0.001, 95%CI [0.21–0.43]), as well as sex of the participant (*r*_*pb*_ *(203)* = 0.42, *p* < 0.001, 95%CI [0.30–0.51]) and chronological age at the 24‐months time‐point (zero‐order; *N* = 206, *r* = 0.19, *p* = 0.008, 95%CI [0.034–0.310]). It did not significantly correlate with SES (*r(203)* = 0.09, *p* = 0.213, 95%CI [−0.07–0.24]) or whether at least one parent spoke Swedish as their first language (*r*_*pb*_(195) = 0.05, *p* = 0.494, 95%CI [−0.11–0.23]), and thus these last two covariates were not included in the following regression analyses.

**TABLE 1:** Descriptive statistics

	Combined typical likelihood group			Elevated likelihood group		
	*N* = 206			*N* = 97		
	M	SD	Range	M	SD	Range
Age at ∼14 Months (days)	438	19	368–501	429	20	389–531
Age at ∼24 Months (days)	756	26	707–919	743	29	660–838
SES	3.3	0.7	1.5–4.0	3.2	0.9	1.0–4.0
Core language composite (14 Months)	0.15	1.85	−3.06 to 5.36	−0.32	1.93	−3.47 to 4.99
Total gesture score (14 Months)	26.13	8.96	3–52	25.51	8.62	0–46
Pragmatics scale (24 Months)	6.36	2.37	0–10	5.07	2.77	0–10
Grammar scale (24 Months)	3.66	3.04	0–12	3.34	3.27	0–12

All three models in the hierarchical regression analysis (Table 2) significantly predicted the Pragmatic Scale and yielded significant *R* ^2^ change (model 1 (control variables): *R* ^2^ = 0.20, *F*(2,203) = 25.52, *p* < 0.001; model 2 (main effects): Δ*R* ^2^ = 0.10, F(2,201) = 14.46, *p* < 0.001; model 3 (interaction term): Δ*R* ^2^ = 0.02, F(1,200) = 4.80, *p* = 0.030). The last model, which included the interaction effect, explained 32% of the total variance. The interaction between the Core Language Composite and the Total Gesture Score was significantly predicting the Pragmatic Scale. Whereas the Core Language Composite was a significant predictor, the Total Gesture Score was not.

**TABLE 2:** Predictors of the pragmatics scale: typical likelihood group

	*b*	Std. Error	95% CI	*t*	*p*
Model 1					
Sex^a^	1.93	0.30	1.35, 2.53	6.51	0.001
Age^b^	0.02	0.01	0.01, 0.02	2.57	0.002
Model 2					
Sex^a^	1.51	0.29	0.94, 2.07	5.16	0.001
Age^b^	0.01	0.01	0.01, 0.02	2.48	0.003
Core language composite	0.69	0.16	0.39, 1.01	4.03	0.001
Total gesture score	0.14	0.16	−0.18, 0.49	0.81	0.386
Model 3					
Sex^a^	1.46	0.29	0.89, 2.03	5.00	0.001
Age^b^	0.01	0.01	0.01, 0.02	2.35	0.006
Core language composite	0.71	0.16	0.41, 1.03	4.19	0.001
Total gesture score	0.16	0.16	−0.16, 0.46	0.92	0.329
Core language composite * total gesture score	−0.28	0.12	−0.53, −0.05	−2.19	0.023

^a^ Reference level: Male.

^b^ Chronological age at the 24‐month time‐point (days).

We followed up the significant interaction effect with a simple slopes moderation analysis, calculating the relation between the Total Gesture Score and the Pragmatics Scale at three values of the Core Language Composite; at mean value, and at one standard deviation above and below the mean. This analysis revealed that the Total Gesture Score predicted the Pragmatics Scale only at low levels of the Core Language Composite (−1 SD: *b* = 0.44, SEM = 0.22, *t* = 1.99, *p* = 0.047, 95%CI [0.01–0.88]; Mean: *b* = 0.16, SEM = 0.17, *t* = 0.92, *p* = 0.359, 95%CI [−0.18–0.50]; +1 SD: *b* = −0.12, SEM = 0.21, *t* = −0.57, *p* = 0.569, 95%CI [−0.54–0.30]). In contrast, for the relation between the Core Language Composite and the Pragmatics Scale on the corresponding three levels of the Total Gesture Score, the Core Language Composite significantly predicted the Pragmatics Scale at all levels of the Total Gesture Score. As a sensitivity analysis we performed the main correlation and regression analyses on only the BATSS sample, excluding the low‐likelihood group from EASE. The results showed that the overall pattern of significance was the same as in the analysis of the combined sample (Appendix S1).

### Link to symptoms and likelihood of ASD

Partial correlations, controlling for chronological age, within the elevated likelihood group showed that, at the 24‐months‐visit, the Pragmatics Scale correlated negatively with the calibrated severity scores of ADOS‐2 Total Score (*r(91)* = −0.36, *p* < 0.001, 95%CI [−.51−0.19]), and of the two subdomains; Social Affect (*r(91)* = −0.35, *p* = 0.001, 95%CI [−0.52−0.16]) and Restrictive and Repetitive Behaviours (*r(91)* = −0.26, *p* = 0.013, 95%CI [−0.46−0.03]). Neither the Total Gesture Score nor the Core Language Composite correlated significantly with the ADOS‐2. To explore the specificity of the association between decontextualized language and autistic symptoms, we performed a regression analysis with the ADOS‐2 Total Score as dependent variable and the Pragmatics Scale, the Grammar Scale, and the Non‐Verbal Developmental Level (NV‐IQ) from Mullen Scales of Early Learning (Mullen, 1995) as predictors. This analysis showed that the association between the Pragmatics Scale and the ADOS‐2 Total Score remained significant even after controlling for concurrent Grammar Scale scores and NV‐IQ (see Appendix S3 and Table S1‐S2 for full description, descriptive statistics and results).

The children in the elevated likelihood group scored significantly lower on the Pragmatics Scale at 24 months compared to the combined typical likelihood group, after controlling for chronological age, with a mean difference of 1.06 points (*F*(1,300) = 11.64, *p* = 0.001). This group difference remained after controlling for concurrent grammatical ability (*F*(1,299) = 22.62, *p* < 0.001). In contrast, the groups did not differ on neither the Core Language Composite (*F*(1,300) = 2.07, *p* = 0.152), the Total Gesture Score (*F*(1,300) = 0.02, *p* = 0.877), or the Grammar Scale (*F*(1,300)<0.01, *p* = 0.984).

To investigate if likelihood of ASD moderated the association between the Total Gesture Score, the Core Language Composite, and the Pragmatics Scale, we entered the three‐way interaction term (Core Language Composite * Total Gesture Score * likelihood group) into a regression model with all lower‐level terms, sex, and chronological age at the 24‐months time‐point. This analysis showed that the three‐way interaction was not significant, indicating that the pattern of prediction did not differ significantly based on likelihood group membership (Table S3). As an exploratory, not pre‐specified analysis we performed the pragmatic scales antecedent analysis on the elevated likelihood group separately. In this analysis there was a main effect of the Core Language Composite, but not the Total Gesture Score, as in the main analysis, but no significant interaction between these to predictors (Appendix S4 and Table S4).

### Secondary analyses

#### Specificity analysis

When the hierarchical regression analysis was repeated with the Grammar Scale as the dependent variable, only the Core Language Composite was a significant predictor, and there was no interaction between the Core Language and Total Gesture Scores (see Appendix S2 for full results). The Pragmatic Scale correlated significantly with the concurrent Grammar Scale (*r(203)* = 0.62, *p* < 0.001, 95%CI [0.54–0.70]).

#### Twin analysis

Descriptive statistics for the twin sample are reported in Table S5. There was a significant effect of sex on both the Grammar Scale (*p* = 0.002) and the Pragmatics Scale (*p* < 0.001).

**FIGURE 1:**
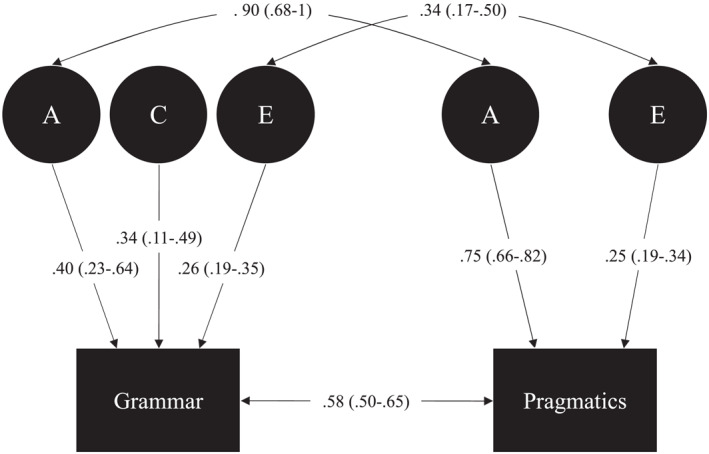
Scheme of the bivariate twin model for the Grammar and Pragmatics Scales at 24 months age. A = genetic influences, C = shared environment influences, E = unique environment influences.

The twin correlations for monozygotic pairs were higher than for dizygotic pairs for the two scales (Pragmatics Scale: MZr = 0.75, 95% CI [0.65–0.82] and DZr = 0.41, 95%CI [0.22–0.56]; Grammar Scale MZr = 0.74, 95%CI [0.65–0.81] and DZr = 0.55, 95%CI [0.37–0.67]), suggesting genetic influences on both. Univariate twin modelling confirmed the genetic contribution to the Grammar and Pragmatics scales, and suggested that shared environment only had an influence on the Grammar Scale (Tables S6‐S9). For this reason, a bivariate model with A, C, and E influences explaining the variation in grammar, and A and E influences explaining the variation in pragmatics, was tested alongside ACE, AE, CE, and E models. The ACE‐AE model was the best fitting model with the lowest AIC statistic. This model did not fit significantly worse than the ACE model, while dropping the C component of the Grammar Scale (the AE model) had a marginally significant poorer fit (Tables S7 and S9). The estimates from the ACE‐AE model showed that the genetic correlation between the two measures was 0.90 (95%CI [0.68–0.1]; Figure 1, Table S10). The upper bound of the CI of 1 indicates the possibility of a complete overlap in the genetics explaining these measures. See Table S11 for test of the assumption of equality of phenotypic and CTCTs correlations across twin order and zygosity.

## DISCUSSION

Our results suggest that compared to early socio‐communicative abilities, early core language abilities have stronger longitudinal associations to later decontextualized language use. However, socio‐communication and core language interacted in predicting decontextualized language, indicating that children with lower levels of early core language might draw from socio‐communicative abilities to compensate for general language deficits in the development of this potential early pragmatic function. This is in line with the hypothesis put forward by Wilson and Bishop (2019, 2021a), proposing that early in development, pragmatics, core language, and socio‐communication may be more closely intercorrelated than for more proficient language users.

As hypothesized, the use of decontextualized language was negatively associated with concurrent autistic symptoms in two‐year‐olds belonging to a group with elevated likelihood of ASD due to having a first‐degree relative with the condition. Neither general non‐verbal developmental level nor concurrent core language abilities could explain this association, which is line with what Wilson and Bishop (2021b) found in adults with ASD. Further, the children with elevated likelihood of ASD used decontextualized language significantly less than the typical‐likelihood children. In contrast, the groups scored similarly on core language and social communication at 14 months as well as grammatical ability at 24 months of age. This was unexpected, given that children with elevated likelihood of ASD has been reported previously to exhibit difficulties with both core language and social communication at this age (Lazenby et al., 2016; Zwaigenbaum et al., 2005). Taken together, these findings suggest that already in toddlerhood, decontextualized language use is independently associated with dimensional aspects of autism as well as with having familial elevated likelihood of developing ASD.

We made two additional explorative analyses to investigate if the developmental antecedents of decontextualized language use extended also to concurrent grammatical language use, and if these two aspects of language use share genetic and environmental aetiology. We found that in contrast to decontextualized language, there was no interaction between core language and social communication in predicting grammatical language ability at 2 years, suggesting a difference in the patterns of antecedents between pragmatic versus core language ability. Pragmatic language (measured here by decontextualized language) and core language (measured by grammatical language) were moderately correlated at 2 years age in our sample (*r* = 0.62), and the twin analyses indicated that most, if not all, of the genetic influence on one aspect were shared with the other. This result suggests that pragmatic and core language are quite strongly associated at this age. On the other hand, the twin analyses also indicated a difference between decontextualized and grammatical language use, in regard to the relative contribution of genetics and shared environment to their respective variances. While shared environment seemed to have an influence on grammar use, this was not the case for the variance in decontextualized language which could be explained solely by genetics and unique environment. Taken together, these results could suggest that even in early development, core language, as measured by grammatical language use, and pragmatics, as measured by decontextualized language use, are associated yet differentiated language domains both on the level of aetiology, developmental antecedents, and behaviour. This is in line with the Relevance Theory, which places pragmatics as a socio‐cognitive capacity in its' own right, separable from core language while still related to it (Carston, 2002; D. Wilson & Sperber, 2002).

Better grammatical ability should facilitate the use of decontextualized language. Conversely, the ability to talk about pragmatically and conceptually advanced phenomena should promote the use of more complex grammar. The Grammar Scale also includes items of both the possessive form and past tense; grammatical structures that are associated with decontextualized language as they are related to talking about things and events beyond the here and now. Our findings that the Pragmatics and Grammar Scales are correlated and that early core language ability might be important for decontextualized language use are therefore not surprising. The fact that we still find differences between these scales in terms of longitudinal prediction and influence of environmental factors suggests that these two scales capture different aspects of early language ability.

The finding that grammatical and decontextualized language use shared almost all of their genetic influence is noteworthy given the clinical association between ASD and pragmatic ability but not necessarily grammatical ability, and the fact that ASD is a highly heritable condition. However, we cannot rule out the possibility of unique genetic influences on either ability, albeit small and non‐significant in our study. Therefore, it is yet possible that there are unique genetic influences on decontextualized language that in turn overlaps with genes associated with ASD. Another possibility, not mutually exclusive, is that the added contribution of shared environment to the variance in grammar use (but not to decontextualized language) plays a role in the distinction seen between these two linguistic constructs and ASD. It is also important to note that while we tested the relative genetic and environmental influences on grammar and pragmatics at 2 years of age, the pattern might change at older ages (Hayiou‐Thomas et al., 2012).

In our study the relationships between core language and social communication was not moderated by likelihood of ASD, indicating that the relative contribution of core language and social communication to decontextualized language use is similar in autistic and typical development. However, when performing the developmental antecedent analysis on the elevated likelihood group separately, we did not find a significant interaction between core language and social communication in predicting decontextualized language use in this group. This is in contrast to what we found for children with typical likelihood of receiving an ASD diagnosis, and could suggest that children with elevated likelihood of ASD may depend less on social communication abilities in the development of decontextualized language. However, because this analysis was explorative, we hesitate to draw strong conclusions regarding this finding.

Pragmatic ability in childhood has been shown to be associated with important social facets of life, not only for children with ASD (Whitehouse et al., 2009). As such, early intervention directed towards improving pragmatic ability might be important not only for children at elevated likelihood of ASD (Klin et al., 2007), but also for the general population. Indeed, variation in early decontextualized language use spanned both our typical likelihood and elevated likelihood groups. Intervention trials are needed in order to understand causal effects, but in order to develop well‐targeted interventions, it is important to first have a detailed understanding of the antecedents of pragmatic abilities. Other individual factors, such as theory of mind, working memory (Zufferey, 2016) or domain‐general inferential processing (A. C. Wilson & Bishop, 2019) may also be important for early pragmatic development, but if decontextualized language is found to be a valid marker of early pragmatic ability, our results indicate also core language as a potentially important target for intervention. Further, based on the interaction between core language and social communication in predicting decontextualized language use, support of social communication could prove beneficial to pragmatic development for young children with delayed or impaired core language abilities.

A potential critique against the decontextualized language measure and our interpretation thereof is that even though decontextualized language may be regarded as a pragmatic aspect later in language development, this is not necessarily the case in early language development when the conversational partner need make contextual interpretation to understand the statement. Three of our findings corroborate the view that decontextualized language use could be regarded as a pragmatic sub‐skill or precursor already this early in development. First, the finding that decontextualized language was associated with dimensional autistic traits, independently from general development and core language. Second, the finding that core language and social communication were differently linked to decontextualized and grammatic language use. Third, the finding that environmental factors differentially influenced decontextualized and grammatic language use. Taken together, these findings suggest that what we measure with decontextualized language captures something beyond core language that is also independently associated with autism, which is what we would expect from a measure of early pragmatic ability. We find this dissociation between decontextualized and core language despite the conceptual similarities between the pragmatics and grammar scales, and despite the fact that both measures are rated by the same parents at the same time, both of which would increase the expected intercorrelation between measures. However, it is a limitation to our study that we only measured one specific early pre‐pragmatic skill, decontextualized language, because there is a lack of previous research validating this measure as specifically pragmatic. To know if our conclusions generalize to other pragmatic abilities we (1) need studies that associate decontextualized language to other pragmatic skills and (2) need to replicate our analysis on other pragmatic skills. This second point, regarding replication, also applies to grammatical ability and socio‐communication, although we measured both semantic and syntactic understanding (in the case of the first) and a rather broad array of social communication abilities (in the case of the second). Further, most of our measures were based on parental report. While this is a limitation for example, due to common method bias and due to difficulty for parents to rate their own children, parental reports also have advantages as they sometimes can give a better picture of certain abilities, such as pragmatics, than what can be captured in formal evaluation (M. Miller et al., 2015). Lastly, there are considerations related to twin births that may challenge the assumption that twins are representative of the general population. In this regard, we excluded participants with developmental concerns, and we discerned no significant differences in our measures between the BATSS sample and the low‐likelihood group from EASE. We therefore conclude that this combined typical‐likelihood group are generally typically developing in the ways relevant for our conclusions, but replication in a non‐twin sample would be advisable in future studies.

## CONCLUSION

We found that for young children with and without elevated likelihood of ASD, core language is a strong predictor of decontextualized language use, and that socio‐communicative abilities may be an important resource for children with lower levels of early general language ability in their development of this potential early pre‐pragmatic language ability. We also found associations between decontextualized language use and autistic symptoms beyond what could be explained by general language ability or developmental level. Further, our results suggests that decontextualized language and core language are phenotypically and genetically associated yet independent aspects of language already early in development. As such, our results add to the understanding of longitudinal and aetiological factors underlying early pragmatic development both in typical and in non‐neurotypical development.

## AUTHOR CONTRIBUTIONS

**Maja Rudling**: Conceptualization; Data curation; Formal analysis; Methodology; Visualization; Writing – original draft. **Ana Maria Portugal**: Data curation; Formal analysis; Methodology; Visualization; Writing – review & editing. **Sven Bölte**: Investigation; Project administration; Resources; Writing – review & editing. **Terje Falck‐Ytter**: Conceptualization; Funding acquisition; Methodology; Project administration; Resources; Supervision; Writing – review & editing.

## CONFLICTS OF INTEREST

The authors have declared no competing or potential conflicts of interest in relation to this article. S.B. discloses that he has in the last 5 years acted as an author, consultant, or lecturer for Medice and Roche. He receives royalties for textbooks and diagnostic tools from Hogrefe (e.g., ADOS‐2, ADI‐R, SRS‐2). He is shareholder in SB Education/Psychological Consulting AB and NeuroSupportSolutions International AB.

### OPEN RESEARCH BADGES

This article has earned a Preregistered Research Designs badge for having a preregistered research design, available at https://doi.org/10.17605/OSF.IO/D6VJS.

## ETHICAL CONSIDERATIONS

BATSS and EASE were approved by the Regional Ethical Board in Stockholm, Sweden and conducted in accordance with the 1964 Declaration of Helsinki. Informed consent was collected from all parents.

## Supporting information

Supporting Information S1

## Data Availability

The data that supports the findings of this study are available from the corresponding author upon reasonable request. Transfer of data will require a data processor agreement according to EU legislation (GDPR). The data are not publicly available because they contain information that could compromise research participant privacy/consent.
