# Supplementary material for: Pre‐pragmatic language use in toddlerhood: Developmental antecedents, aetiological factors, and associations to autism
Source: JCPP Adv. 2023 Jan 11;3(1):e12135. doi: 10.1002/jcv2.12135 (PMC10241459; doi:10.1002/jcv2.12135)
Supplement: Supplementary file 1 — Supporting Information S1 [file JCV2-3-e12135-s001.docx]

Supporting Information

**Pre-pragmatic language use in toddlerhood: Developmental antecedents, etiological factors, and associations to autism**

**Appendix S1**

***Sensitivity Analysis using only the BATSS sample***

As a sensitivity analysis we performed the main correlation and regression analyses on only the BATSS sample, excluding the low-likelihood group from EASE. The results showed that the overall pattern of significance was the same as in the analysis of the combined sample, with the exception of chronological age at the 24-month time point which was non-significant in this sample (zero-order; *N* = 178, *r* = .13, *p* = .096, BCa 95% CI [-.04, 0.26]). In contrast to the combined typical likelihood sample, SES significantly correlated with the Pragmatics Scale in the BATSS sample (*r(175)* = .15, *p* = .041, BCa 95% CI [-.03, .29]). However, including SES as a control variable in the regression analysis did not change the overall pattern of significance. One participant in the BATSS sample had a reported family member with an ASD diagnosis. Removing this participant from the analyses did not change the significance of effects.

**Appendix S2**

**Specificity Analysis: *Predictors of the Grammar Scale for the typical likelihood group***

We conducted the same hierarchical regression analysis as we did for the Pragmatics Scale but with the Grammar Scale as outcome variable: 1) Control variables, 2) The Core Language Composite and Total Gesture Score, 3) The interaction term. All three models significantly predicted the Grammar Scale, but the last model, which included the interaction term, did not yield significant R^2^ change (model 1 (control variable): R^2^ = .17, *F*(2,203) = 20.26, *p* < .001; model 2 (main effects): ΔR^2^ = .08, F(2,201) = 10.62 , p < .001; model 3 (interaction effect): ΔR^2^ < 0.01, F(1,200) = 0.02, p = .897). Accordingly, the interaction term between the Core Language Composite and Total Gesture Score was not significant (*b* = -0.02, SEM = 0.17, *t* = -0.13, *p* = .887, BCa 95% CI [-0.33, 0.28]). Similarly to the Pragmatic Scale, however, the Core Language Composite was a significant predictor of the Grammar Scale (*b* = 0.75, SEM = 0.24, *t* = 3.28, *p* = .003, BCa 95% CI [0.27, 1.23]) whereas the Total Gesture Score was not significant (*b* = 0.22, SEM = 0.24, *t* = 0.95, *p* = .351, 95% CI [-0.23, 0.66]).

**Appendix S3**

***Specificity of Associations Between The Pragmatics Scale And ADOS-2***

To explore the specificity of the association between decontextualized language and autistic symptoms at 24 months, we performed correlation and regression analyses of the association between the Pragmatics Scale and ADOS-2 Total Score, including the concurrent Grammar Score from the SECDI and the Non-Verbal Developmental Score (NV-IQ) from the Mullen Scales of Early Learning, MSEL, as control variables. The MSEL is a standardized assessment of young children’s developmental level. The NV-IQ includes the subscales fine motor and visual reception, and was included to test if general developmental level would explain the association between decontextualized language use and autistic symptoms (excluding Verbal Developmental Level, to avoid overlap with the predictors already included in the model).

The ADOS-2 Total Calibrated Severity Score (ADOS-2 Total Score) correlated significantly with the Pragmatics Scale (*r* (94) = -.36, *p* = <.001, 95% CI [-.53, -.17]), the Grammar Scale (*r* (94) = -.29, *p* = .005, 95% CI [-.46, -.09]), and the NV-IQ score (*r* (92) = -.30, *p* = .004, 95% CI [-.47, -.10]). However, the ADOS-2 Total Score did not correlate with sex (*r_pb_* (94) = -.12, *p* = .259, BCa 95% CI [-.31, .09]) in the elevated-likelihood group, and sex was thus not included as a covariate in the following regression analysis.

A hierarchical regression analysis was performed, with the ADOS-2 Total Score as dependent variable, entering predictors in the following order: 1) The Pragmatics Scale 2) Grammar Score and NV-IQ. Both models significantly predicted the ADOS-2 Total Score (**Table S1**), but the second model, including all predictors, did not yield significant R^2^ change (ΔR^2^=.04, F(2,88)=1.86, p=.162). Neither the Grammar Scale nor NV-IQ was significant predictors in that model, whereas the Pragmatics Scale was (see **Table S2** for full results of this analysis).

**Appendix S4**

***Pragmatic scale antecedent analysis on the elevated likelihood group***

As for the combined typical likelihood group, the Pragmatic Scale correlated with the Core Language Composite score (*r* (94) = .38, *p* < .001 , BCa 95% CI [.17, .56]), the Total Gesture Score (*r* (94) = .34, *p* = .001, BCa 95% CI [.16, .52]), and chronological age at the 24-month time-point (zero-order; *N* = 97, *r* = .22, *p* = .034, BCa 95% CI [.01, .41]), but not significantly with SES (*r (94)* = .10, *p* = .346, BCa 95% CI [-.08, .29]) or with whether any parent spoke Swedish as their first language (*r (54)* = -.08, *p* = .55, BCa 95% CI [-.36, .19]). However, unlike in the typical likelihood group, the Pragmatic Scale did not correlate with sex (*r* (94) = .15, *p* = .135, BCa 95% CI [-.04, .33]) in the elevated likelihood group.

In the elevated likelihood group, the first two models of the hierarchical regression analysis (**Table S1**) significantly predicted the Pragmatic Scale and yielded significant R^2^ change (model 1 (control variables): R^2^ = .07, *F*(2,94) = 3.49, *p* = .035; model 2 (main effects): ΔR^2^ = .14, F(2,92) = 8.32 , p < .001). Model 2, which included control variables and the two predictors, explained 21% of the variance of the Pragmatic Scale. However, although model 3, which included the interaction effect, was over-all significant (p < .001), it did not yield significant R^2^ change (ΔR^2^ < .01, F(1,91) = 0.26, p = .614). Correspondingly, the interaction between the Core Language Composite and the Total Gesture Score was not significant. In both model 2 and model 3, the Core Language Composite significantly predicted the score of the Pragmatics Scale, whereas the Total Gesture Score did not. Despite the non-significant correlation between sex and the Pragmatics Scale in the elevated likelihood group, sex was added as a control variable in the hierarchical regression analysis to make results between groups more comparable. The over-all results of the regression analysis did not differ when excluding sex as a covariate.


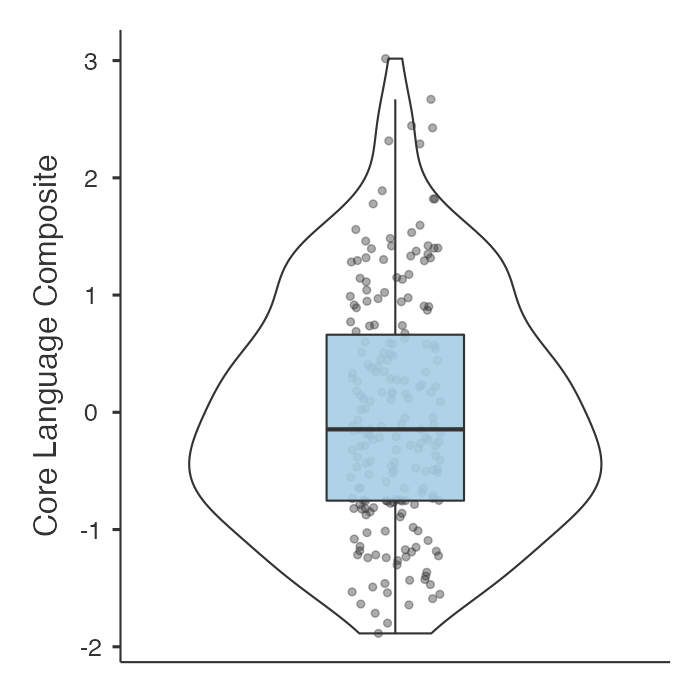


**Figure S1.** Distribution of the Core Language Composite with age regressed out, for the combined typical likelihood group.


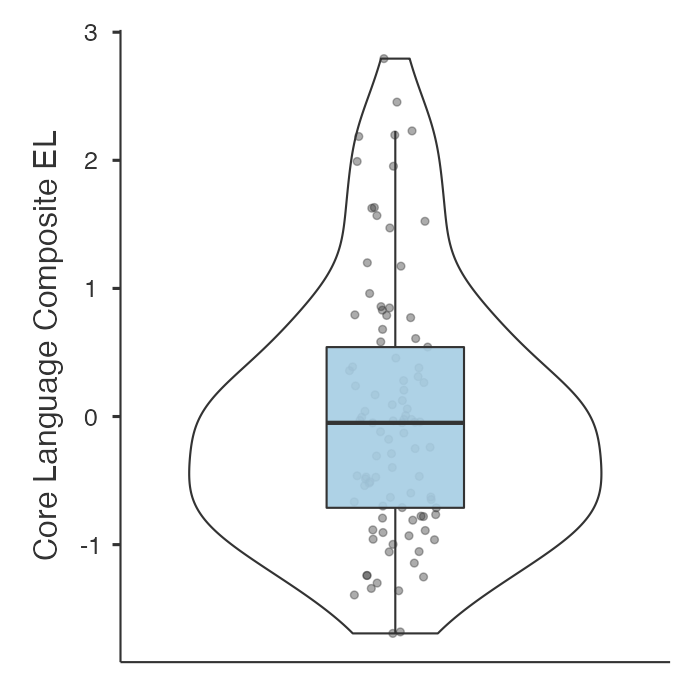


**Figure S2.** Distribution of the Core Language Composite with age regressed out, for the elevated likelihood group.


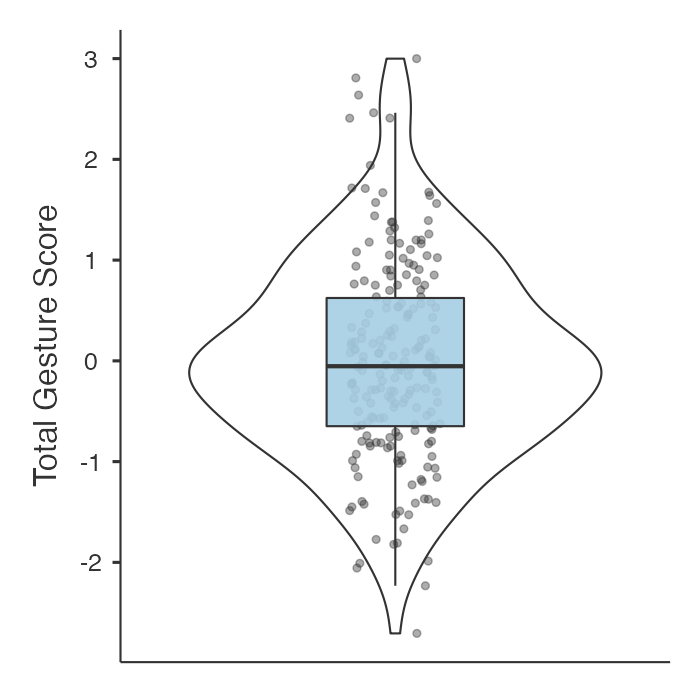


**Figure S3.** Distribution of the Total Gesture Score with age regressed out, for the combined typical likelihood group.


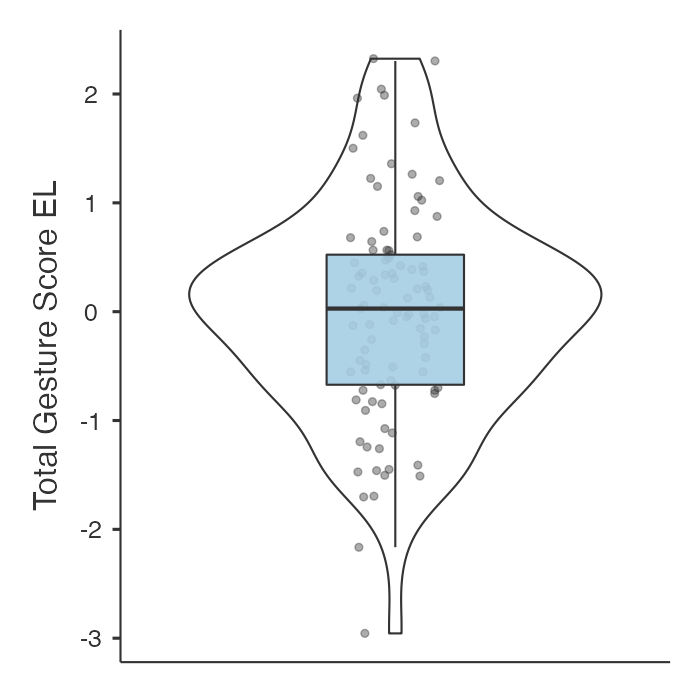


**Figure S4.** Distribution of the Total Gesture Score with age regressed out, for the elevated likelihood group.


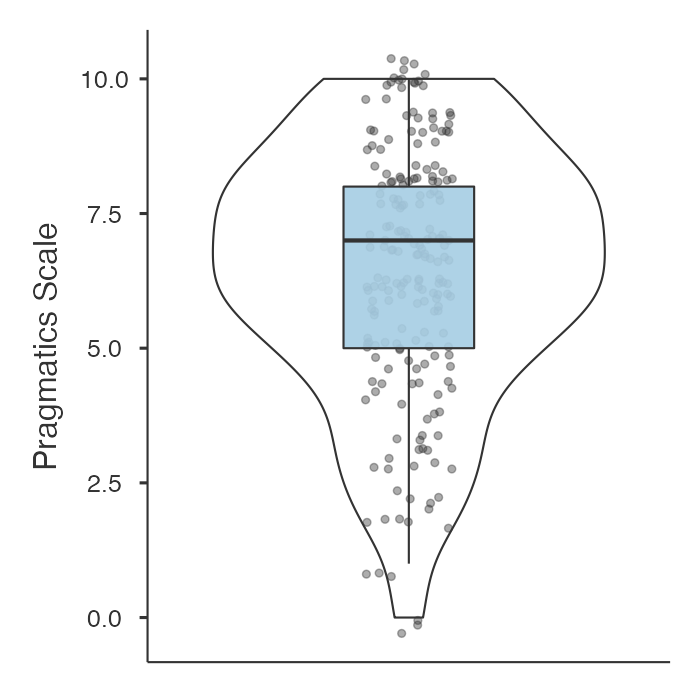


**Figure S5.** Distribution of the Pragmatic Scale, for the combined typical likelihood group.


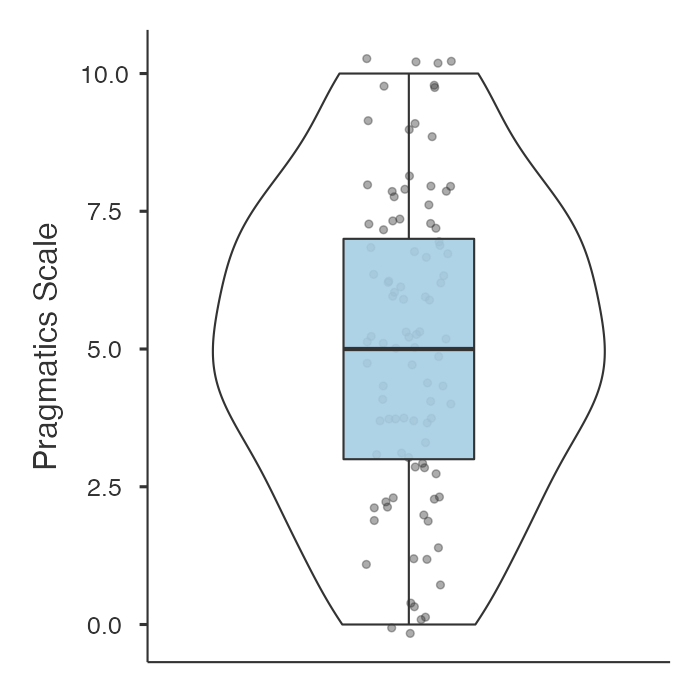


**Figure S6.** Distribution of the Pragmatic Scale, for the elevated likelihood group.


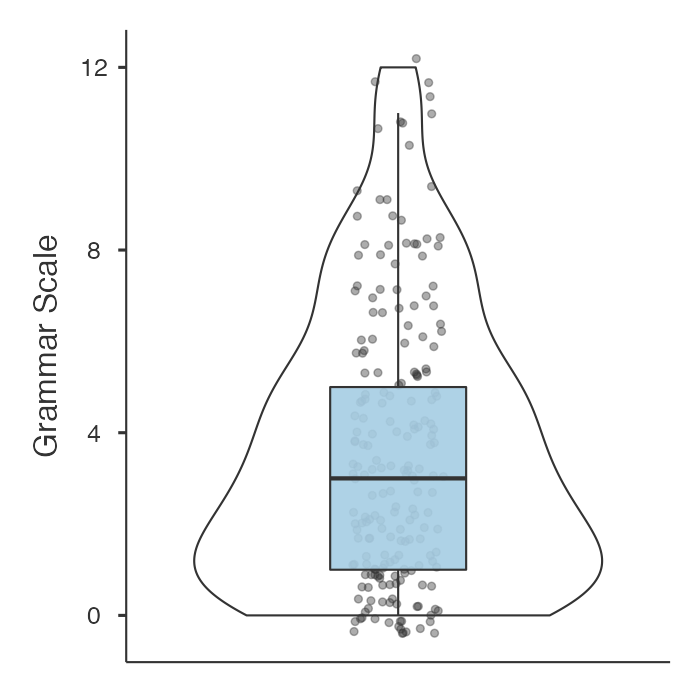


**Figure S7.** Distribution of the Grammar Scale, for the combined typical likelihood group.

**Table S1. ADOS-2 and MSEL for the EASE sample divided into likelihood groups**

|  | **EASE Elevated Likelihood N = 27** | | | |  | **EASE Typical Likelihood *N = 94*** | | | |
| --- | --- | --- | --- | --- | --- | --- | --- | --- | --- |
|  |  |  |  |  |  |  |  |  |  |
|  | ***M*** | ***SD*** | ***Range*** | |  | ***M*** | ***SD*** | ***Range*** | |
|  |  |  | ***Lower*** | ***Upperr*** |  |  |  | ***Lower*** | ***Upper*** |
| ***ADOS-2 Total Score CS*** | 3.74 | 2.19 | 1 | 9 |  | 2.48 | 1.75 | 1 | 8 |
| ***ADOS-2 Social Affect CS*** | 3.82 | 2.02 | 1 | 10 |  | 3.07 | 2.02 | 1 | 10 |
| ***ADOS-2 Restrictive and Repetitive Behaviours CS*** | 5.37 | 1.79 | 1 | 9 |  | 3.85 | 2.09 | 1 | 6 |
|  |  |  |  |  |  |  |  |  |  |
| ***MSEL Early Learning Composite Score*** | 96.99 | 14.82 | 50.00 | 136.00 |  | 108.52 | 13.55 | 64.00 | 132.00 |
| ***MSEL Non-verbal Developmental level (NVIQ)*** | 99.51 | 10.87 | 66.00 | 140.00 |  | 108.07 | 9.67 | 84.00 | 140.00 |
| ***MSEL Verbal Developmental Level (VIQ)*** | 98.66 | 18.72 | 32.00 | 138.00 |  | 109.00 | 17.24 | 50.00 | 135.00 |

| **Table S2: Specificity of Associations Between The Pragmatics Scale And ADOS-2: Regression analysis with ADOS-2 Calibrated Severity Score As Dependent Variable** | | | | | | | |
| --- | --- | --- | --- | --- | --- | --- | --- |
|  | |  | b | Std. Error | 95 % CI | t | p |
| **Model 1** | | |  |  |  |  |  |
|  | Pragmatics Scale | | -0.28 | 0.08 | -0.44, -0.13 | -3.64 | <.001 |
| **Model 2** | | |  |  |  |  |  |
|  | Pragmatics Score | | -0.21 | 0.10 | -0.42, -0.01 | -2.13 | 0.036 |
|  | Grammar Score | | -0.02 | 0.09 | -0.20, 0.16 | -0.24 | 0.813 |
|  | MSEL NV-IQ | | -0.04 | 0.02 | -0.08, 0.00 | -1.79 | 0.076 |
| Model 1: R^2^ = .13, F (1, 90) = 13.22, p < .001  Model 2: R^2^ = .16, F (3, 88) = 5.73, p = .001 | | | | | | | |

| **Table S3. Predictors of The Pragmatics Scale – Analysis of Likelihood Group interaction** | | | | | | |
| --- | --- | --- | --- | --- | --- | --- |
|  |  | b | Std. Error | BCa 95 % CI | t | p |
|  | Sex | 1.09 | 0.28 | 0.49, 1.67 | 4.13 | .001 |
|  | Age (24-month time point; days) | 0.01 | 0.004 | 0.004, 0.02 | 2.73 | .003 |
|  | Core Language Composite | 0.75 | 0.17 | 0.42, 1.07 | 3.94 | .001 |
|  | Total Gesture Score | 0.22 | 0.16 | -0.05, 0.50 | 1.17 | .170 |
|  | Group | -1.20 | 0.35 | -1.91, -0.44 | -3.84 | .002 |
|  | Core language Composite *  Total Gesture Score | -0.30 | 0.12 | -0.56, -0.02 | -2.10 | .015 |
|  | Core language Composite * Group | -0.25 | 0.41 | -1.07, 0.59 | -0.74 | .532 |
|  | Total Gesture Score * Group | 0,37 | 0.42 | -0.39, 1.24 | 1.08 | .359 |
|  | Core language Composite *  Total Gesture Score * Group | 0.41 | 0.28 | -0.14, 0.93 | 1.65 | .129 |
| R^2^ = .31, F (9, 293) = 14.31, p < .001 | | | | | | |

| **Table S4: Predictors of the Pragmatics Scale – Elevated Likelihood Group** | | | | | | | |
| --- | --- | --- | --- | --- | --- | --- | --- |
|  | |  | b | Std. Error | BCa 95 % CI | t | p |
| **Model 1** | | |  |  |  |  |  |
|  | Sex | | 0.83 | 0.55 | -0.27, 1.87 | 1.51 | .132 |
|  | Age 24 Months (days) | | 0.02 | 0.01 | 0.00, 0.04 | 2.13 | .038 |
| **Model 2** | | |  |  |  |  |  |
|  | Sex | | 0.33 | 0.50 | -0.66, 1.29 | 0.62 | .525 |
|  | Age 24 Months (days) | | 0.01 | 0.01 | -0.01, 1.09 | 1.31 | .205 |
|  | Core Language Composite | | 0.74 | 0.36 | -0.04, 1.58 | 2.20 | .039 |
|  | Total Gesture Score | | 0.50 | 0.37 | -0.16, 1.09 | 1.57 | .182 |
| **Model 3** | | |  |  |  |  |  |
|  | Sex | | 0.30 | 0.51 | -0.76, 1.30 | 0.56 | .561 |
|  | Age 24 Months (days) | | 0.01 | 0.01 | -0.01, 0.03 | 1.32 | .199 |
|  | Core Language Composite | | 0.70 | 0.39 | -0.13, 1.68 | 2.03 | .077 |
|  | Total Gesture Score | | 0.50 | 0.38 | -0.17, 1.11 | 1.56 | .183 |
|  | Core language Composite *  Total Gesture Score | | 0.12 | 0.24 | -0.33, 0.55 | 0.51 | .576 |

**Table S5. Descriptive statistics of the twin analysis sample. Statistics presented as Mean (SD) / min-max. MZ, monozygotic; DZ, dizygotic.**

|  | **Overall** | **MZ Females** | **MZ Males** | **DZ Females** | **DZ Males** | **Skewness** |
| --- | --- | --- | --- | --- | --- | --- |
| **N twins** | 374* | 98 | 111 | 92 | 73 |  |
| **Age (in days)** | 756.39 (23.73)  707–920 | 755.71 (18.7)  729–807 | 756.19 (19.88)  707–805 | 755.79 (33.13)  726–920 | 758.37 (21.18)  723–844 | 2.46 |
| **Pragmatics** | 5.99 (2.54)  0–10 | 7.11 (2.14)  0–10 | 5.06 (2.55)  0–10 | 6.59 (1.99)  0–10 | 5.16 (2.83)  0–10 | -0.49 |
| **Grammar** | 3.29 (2.97)  0–12 | 4.04 (3.02)  0–12 | 2.45 (2.9)  0–12 | 3.79 (2.93)  0–12 | 2.9 (2.69)  0–10 | 1.03 |

*198 pairs

**Table S6. Univariate saturated model for grammar including sex as a covariate.**

|  |  |  |  |  | **Comparative fit with saturated model** | | |
| --- | --- | --- | --- | --- | --- | --- | --- |
| **Model** | **# Parameters** | **-2LL** | ***df*** | **AIC** | **Δ χ2** | **Δ *df*** | **P value** |
| Saturated | 11 | 929.97 | 363 | 203.97 | NA | NA | NA |
| Sex | 10 | 939.23 | 364 | 211.23 | 9.25 | 1 | 0.002 |
| 1. | 9 | 932.55 | 365 | 202.55 | 2.58 | 2 | 0.276 |
| 2. | 8 | 932.93 | 366 | 200.93 | 2.95 | 3 | 0.399 |
| 3. | 6 | 934.44 | 368 | 198.44 | 4.47 | 5 | 0.484 |
| 4. | 5 | 935.13 | 369 | 197.13 | 5.16 | 6 | 0.524 |

Model definitions. The baseline model is the fully saturated model of the observed data, which models the means and variances separately for each twin in a pair and across zygosity. **Age.** Testing the significance of age, **Sex.** Testing the significance of sex, **1.** Equating means across twins within a pair, **2.** Equating means across zygosity, **3.** Equating variances across twins within a pair, and **4.** Equating variances across zygosity (i.e., the constrained saturated model).

-2LL = fit statistic, which is minus two times the log-likelihood of the data.

*df* = degrees of freedom

AIC, an alternative fit index. Lower values denote better model fits.

Δ χ2 = difference in −2LL statistic between two models, distributed χ2.

Δ *df =* difference in degrees of freedom between two models.

**Table S7. Univariate twin model fit statistics and parameter estimates for grammar. Best-fitting model in bold.**

| Model | #  Para-meters | -2LL | *df* | AIC | Compar-ison  model | Δ χ2 | Δ *df* | P value | A | C | E |
| --- | --- | --- | --- | --- | --- | --- | --- | --- | --- | --- | --- |
| Fully Sat | 11 | 929.97 | 363 | 203.97 | NA | - | - | - | - | - | - |
| ACE | **5** | **935.13** | **369** | **197.13** | **Fully Sat.** | **5.16** | **6** | **0.524** | **.39 (.10-.76)** | **.34 (0-.60)** | **.26 (.19-.36)** |
| AE | 4 | 938.67 | 370 | 198.67 | ACE | 3.54 | 1 | 0.060 | .74 | 0 | .26 |
| CE | 4 | 942.30 | 370 | 202.30 | ACE | 7.17 | 1 | 0.007 | 0 | .66 | .34 |
| E | 3 | 1045.21 | 371 | 303.21 | ACE | 110.08 | 2 | 0.000 | 0 | 0 | 1 |

-2LL = fit statistic, which is minus two times the log-likelihood of the data.

*df* = degrees of freedom

AIC, an alternative fit index. Lower values denote better model fits.

Δ χ2 = difference in −2LL statistic between two models, distributed χ2.

Δ *df =* difference in degrees of freedom between two models.

**Table S8. Univariate saturated model for pragmatics including sex as a covariate.**

|  |  |  |  |  | **Comparative fit with saturated model** | | |
| --- | --- | --- | --- | --- | --- | --- | --- |
| **Model** | **# Parameters** | **-2LL** | ***df*** | **AIC** | **Δ χ2** | **Δ *df*** | **P value** |
| Saturated | 11 | 920.73 | 363 | 194.73 | - | - | - |
| Sex | 10 | 946.29 | 364 | 218.29 | 25.56 | 1 | < 0.001 |
| 1. | 9 | 923.34 | 365 | 193.34 | 2.61 | 2 | 0.272 |
| 2. | 8 | 925.63 | 366 | 193.63 | 4.90 | 3 | 0.179 |
| 3. | 6 | 927.76 | 368 | 191.76 | 7.02 | 5 | 0.219 |
| 4. | 5 | 927.95 | 369 | 189.95 | 7.22 | 6 | 0.301 |

Model definitions. The baseline model is the fully saturated model of the observed data, which models the means and variances separately for each twin in a pair and across zygosity. **Age.** Testing the significance of age, **Sex.** Testing the significance of sex, **1.** Equating means across twins within a pair, **2.** Equating means across zygosity, **3.** Equating variances across twins within a pair, and **4.** Equating variances across zygosity (i.e., the constrained saturated model).

-2LL = fit statistic, which is minus two times the log-likelihood of the data.

*df* = degrees of freedom

AIC, an alternative fit index. Lower values denote better model fits.

Δ χ2 = difference in −2LL statistic between two models, distributed χ2.

Δ *df =* difference in degrees of freedom between two models.

**Table S9. Univariate twin model fit statistics and parameter estimates for pragmatics. Best-fitting model in bold.**

| Model | #  Para-meters | -2LL | *df* | AIC | Compar-ison  model | Δ χ2 | Δ *df* | P value | A | C | E |
| --- | --- | --- | --- | --- | --- | --- | --- | --- | --- | --- | --- |
| Fully Sat | 11 | 920.73 | 363 | 194.73 | NA | - | - | - | - | - | - |
| ACE | 5 | 927.95 | 369 | 189.95 | Fully Sat. | 7.22 | 6 | 0.301 | .68 | .06 | .25 |
| AE | **4** | **928.08** | **370** | **188.08** | **ACE** | **0.13** | **1** | **0.719** | **.75 (.66-.82)** | **0** | **.25 (.18-.34)** |
| CE | 4 | 945.43 | 370 | 205.43 | ACE | 17.48 | 1 | < 0.001 | 0 | .59 | .41 |
| E | 3 | 1023.63 | 371 | 281.63 | ACE | 95.67 | 2 | < 0.001 | 0 | 0 | 1 |

-2LL = fit statistic, which is minus two times the log-likelihood of the data.

*df* = degrees of freedom

AIC, an alternative fit index. Lower values denote better model fits.

Δ χ2 = difference in −2LL statistic between two models, distributed χ2.

Δ *df =* difference in degrees of freedom between two models.

**Table S10. Bivariate twin model fit statistics for grammar and pragmatics. Best-fitting model in bold.**

| Model | # Parameters | -2LL | *df* | AIC | Comparison model | Δ χ2 | Δ *df* | P value |
| --- | --- | --- | --- | --- | --- | --- | --- | --- |
| Fully Sat | 30 | 1710.52 | 718 | 274.52 | NA | NA | NA | NA |
| ACE |  |  |  |  |  |  |  |  |
| ACE | 13 | 1728.26 | 735 | 258.26 | Fully Sat. | 17.74 | 17 | 0.405 |
| ACE nested models | |  |  |  |  |  |  |  |
| AE | 10 | 1736.01 | 738 | 260.01 | ACE | 7.76 | 3 | 0.051 |
| CE | 10 | 1748.74 | 738 | 272.74 | ACE | 20.48 | 3 | < 0.001 |
| E | 7 | 1915.79 | 741 | 433.79 | ACE | 187.53 | 6 | < 0.001 |
| ACE-AE | **11** | **1728.42** | **737** | **254.42** | **ACE** | **0.16** | **2** | **0.922** |

Model definitions. The Fully Sat. model is the fully saturated model of the observed data, which models the means and variances for both variables, and the phenotypic and cross-twin-cross-trait correlations between the two variables, separately for each twin in a pair and across zygosity.

-2LL = fit statistic, which is minus two times the log-likelihood of the data.

*df* = degrees of freedom

AIC, an alternative fit index. Lower values denote better model fits.

Δ χ2 = difference in −2LL statistic between two models, distributed χ2.

Δ *df =* difference

**Table S11. Assumptions testing for the bivariate model between grammar and pragmatics.**

|  |  |  |  |  | **Comparative fit with saturated model** | | |
| --- | --- | --- | --- | --- | --- | --- | --- |
| **Model** | **# Parameters** | **-2LL** | ***df*** | **AIC** | **Δ χ2** | **Δ *df*** | **P value** |
| Saturated | 30 | 1710.52 | 718 | 274.52 | - | - | - |
| 5. | 13 | 1728.26 | 735 | 258.26 | 17.74 | 17 | 0.405 |

Model definitions. The Fully Sat. model is the fully saturated model of the observed data, which models the means and variances for both variables, and the phenotypic and cross-twin-cross-trait correlations between the two variables, separately for each twin in a pair and across zygosity. **5.** In the Bivariate model fitting, the constrained saturated model equates means, variances, phenotypic and cross-twin-cross-trait correlations across twins within a pair and across zygosity, for both variables of interest.

The best-fitting model (in bold) was the non-significant and most parsimonious model, as well as the one with the lowest AIC.

-2LL = fit statistic, which is minus two times the log-likelihood of the data.

*df* = degrees of freedom

AIC, an alternative fit index. Lower values denote better model fits.

Δ χ2 = difference in −2LL statistic between two models, distributed χ2.

Δ *df =* difference
